# Cell Reporter-Based Analysis of Anti-Inflammasome Activities of Flavonoids with Distinct Structures

**DOI:** 10.3390/molecules31183176

**Published:** 2026-09-10

**Authors:** Nora Pierce, Christopher Schulman, Vijay Yechoor, Ke Ma, Young-Hwa Goo, Antoni Paul

**Affiliations:** 1 Department of Molecular and Cellular Physiology, Albany Medical College, 43 New Scotland Ave, Albany, NY 12208, USA; pierceno@amc.edu (N.P.); schulmc@amc.edu (C.S.); gooy@amc.edu (Y.-H.G.); 2 Division of Endocrinology and Metabolism, Diabetes and Beta Cell Biology Center, Department of Medicine, University of Pittsburgh, Pittsburgh, PA 15213, USA; yechoorv@pitt.edu; 3 Department of Diabetes Complications and Metabolism, Beckman Research Institute of City of Hope, Duarte, CA 91010, USA; kema@coh.org

**Keywords:** cell reporter, flavonoids, functional assay, inflammasome, inflammation, interleukin-1beta, macrophages

## Abstract

Flavonoids are natural compounds that have shown promise against chronic inflammation. Despite sharing a common core scaffold, flavonoids exhibit substantial structural heterogeneity, and comparisons of biological activities across subclasses remain limited. These studies are often constrained by the cost and sensitivity limitations of conventional analytical approaches. The main objective of this study was to validate a cell-based reporter system, HEK-Blue IL-1R cells, as an alternative to immunoassays for assessing flavonoid structure–activity relationships on the modulation of inflammasome-driven IL-1β secretion by macrophages. Under mild inflammatory stimulation with lipopolysaccharide (LPS), the reporter displayed sensitive and efficient detection of low-level IL-1β signaling. In a comparative analysis of three flavonoids differing in polarity and glycosylation, quercetin, a polar aglycone, and nobiletin, a methoxylated aglycone, significantly inhibited reporter responses relative to LPS-stimulated controls, with a trend toward greater inhibition detected for quercetin. In contrast, no inhibitory effect was detected for rutin, a glycosylated derivative of quercetin. These findings were consistent with the expression of NLRP3 inflammasome proteins responsible for IL-1β maturation and secretion. Collectively, these results support the potential of flavonoid aglycones against inflammasome-associated inflammation and highlight the HEK-Blue IL-1R reporter system as a sensitive and cost-effective platform for screening IL-1β signaling under conditions of low-grade inflammation.

## 1. Introduction

Low-grade inflammation contributes to the pathogenesis of many chronic, noncommunicable diseases. While acute inflammation is a protective mechanism of the innate immune system to eliminate pathogens and promote tissue repair, systemic chronic inflammation often results from homeostatic imbalances unrelated to host defense and can lead to the damage of healthy tissues [1]. Tissue dysfunction and stress stimuli across diverse physiological systems can initiate an inflammatory response, as evidenced by persistent inflammation associated with conditions such as atherosclerosis, cancer, insulin resistance, and Alzheimer’s disease [2,3,4,5]. If not controlled, inflammation can further exacerbate these diseases. Although anti-inflammatory medications such as non-steroidal anti-inflammatory drugs (NSAIDs) and corticosteroids are widely used, their long-term use is associated with significant adverse effects [6,7].

The NLRP3 inflammasome is a key component of the innate immune system and its activation contributes to the development of a broad range of chronic diseases [8,9,10,11]. Inflammasome activation involves two steps. Under inflammatory conditions, cells respond to damage- or pathogen-associated molecular patterns (DAMPs/PAMPs), cytokines, or other stimuli that act as priming signals. These signals induce the expression of inflammasome components as well as pro-IL-1β and pro-IL-18 through receptor-mediated NF-κB activation [12,13,14,15]. The second step involves oligomerization and activation of the NLRP3 components into functional inflammasomes in response to stimuli such as cellular stress, dysregulated calcium signaling, or lipid factors [16,17,18]. Upon the assembly of NLRP3 with pro-caspase-1 and apoptosis-associated speck-like protein containing a CARD (ASC), pro-caspase-1 is cleaved into its active form, enabling the processing of pro-IL-1β and pro-IL-18 and secretion of the mature, bioactive cytokines [15,16,17,18]. Thus, bioactive IL-1β signaling serves as a direct functional biomarker for assessing inflammasome activation and the efficacy of potential inhibitors.

Flavonoids are natural polyphenolic compounds found in fruits, vegetables, cereals, and nuts, and are commonly included in dietary supplements. They exhibit a wide range of therapeutic properties, including the inhibition of inflammasome and broader anti-inflammatory effects that may help mitigate chronic low-grade inflammation [19,20,21,22]. Structurally, flavonoids share a characteristic 3-ring scaffold, with 15 carbons arranged in two aromatic rings connected by a central pyrone ring [23,24]. Despite the common core scaffold, there is significant structural heterogeneity that could affect the bioactivity of these compounds. Two of the most prevalent flavonoid classes are flavones and flavonols, both characterized by a double bond between positions 2 and 3 of the central ring [23,24]. Flavonols contain a characteristic hydroxyl group at position 3 of the central ring in addition to variable hydroxyl groups on the A and B rings that confer polarity and enable O-glycosidic linkage with sugars. In contrast, flavones generally lack this 3-hydroxyl group. Citrus-derived flavones such as nobiletin and tangeretin are particularly rich in methoxy groups, rendering them less polar and less amenable to glycosylation [23,24,25]. Although both hydroxy and methoxy flavones have been shown to inhibit the NLRP3 inflammasome, the impact of these structural differences on the production of mature, functional, IL-1β in the context of low-grade inflammatory conditions remains poorly understood [22,26,27,28,29].

**Figure 1:**
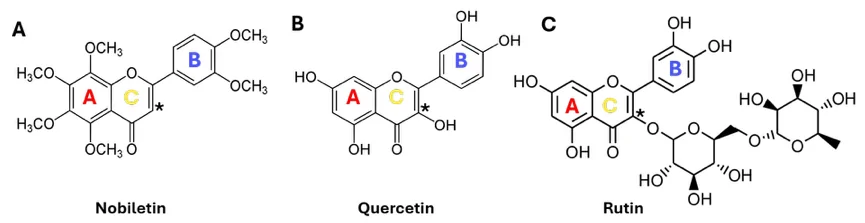
Molecular structures of nobiletin (**A**), quercetin (**B**), and rutin (**C**). Benzene rings are labeled A and B, and the pyrone ring is labeled C. Carbon 3 of ring C is indicated by an asterisk (*), reflecting the key structural difference between flavones and flavonols.

Conventional assays to detect IL-1β, such as enzyme-linked immunosorbent assays (ELISAs), typically offer broad dynamic ranges but may lack the sensitivity to resolve subtle cytokine changes associated with low-grade inflammatory stimuli [30,31,32]. While ultrasensitive ELISAs and other high-sensitivity platforms can address this limitation, these approaches can become time- and cost-prohibitive when analyzing large sample sizes [33]. As an alternative, this study evaluated a HEK-Blue reporter cell line that stably expresses the human IL-1 receptor (IL-1R) coupled with an NF-κB/AP-1-inducible secreted embryonic alkaline phosphatase (SEAP) reporter [34,35,36]. Unlike conventional immunoassays, which quantify cytokine protein concentrations and may detect both precursor and mature cytokine forms depending on antibody specificity, the HEK-Blue system measures functional IL-1R activation. As a result, SEAP production and secretion into the culture medium reflects biologically active IL-1R signaling, providing a highly sensitive approach that can be leveraged as a high-throughput platform for assessing inflammasome activation [37,38]. Three flavonoids were selected based on structural features to compare their ability to inhibit NLRP3 activation. Nobiletin (NOB; Figure 1A) is a polymethoxylated flavone with methoxy groups on rings A and B. Quercetin (QUER; Figure 1B) is a flavonol with polar hydroxyl groups distributed across rings A, B, and C. Rutin (RUT; Figure 1C) is a glycosylated derivative of quercetin, with an O-glycosidic linkage of a glucose-rhamnose disaccharide to ring C. Both aglycones significantly inhibited SEAP production in response to LPS challenge, with the more polar QUER exhibiting slightly higher inhibition. In contrast, RUT had no detectable effect on SEAP production. The HEK-Blue reporter findings were consistent with immunoblot analysis of inflammasome-associated proteins, supporting the utility of this approach for screening compounds with potential anti-inflammasome activity under conditions of low-level inflammatory signaling.

## 2. Results

### 2.1. IL-1β Detection Approaches

In previous studies, we found that treatment of mouse bone marrow-derived macrophages (BMMs) with lipopolysaccharide (LPS; 10 ng/mL, 24 h) was sufficient to activate the NLRP3 inflammasome and induce moderate IL-1β secretion [30]. Although the relatively low levels of IL-1β produced under these conditions may better reflect those seen under common chronic conditions, such as metabolic and cardiovascular diseases, they pose technical challenges for evaluating protective interventions that reduce IL-1β secretion. To address this limitation, we compared a high-sensitivity, bioluminescence-based immunoassay with a cell-based IL-1 receptor (IL-1R) reporter system.

#### 2.1.1. Bioluminescence-Based Immunoassay

**Figure 2:**
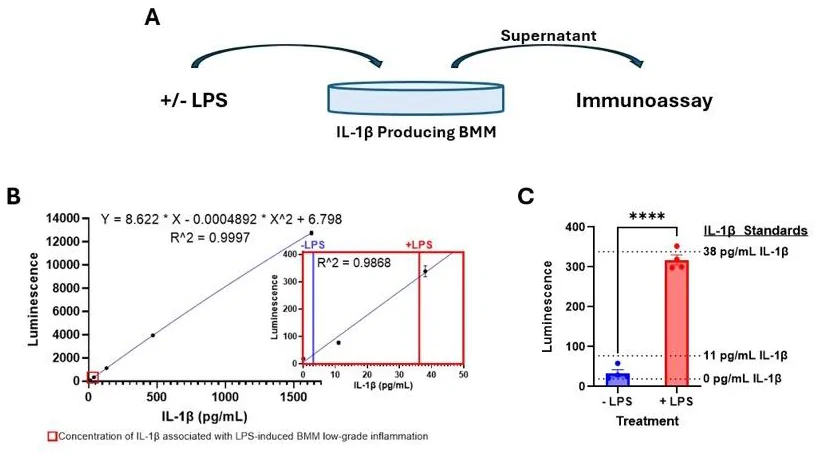
Evaluation of the bioluminescence-based immunoassay for IL-1β detection. (**A**) Experimental workflow for generating IL-1β-containing supernatants from BMMs treated in the presence or absence of LPS (10 ng/mL, 24 h). (**B**) Nonlinear regression model generated using IL-1β standards ranging from 0–1630 pg/mL. The red box highlights the concentration range associated with low-grade inflammation (0–50 pg/mL). The +LPS and −LPS lines indicate mean IL-1β levels in supernatants from BMM that remained untreated or were treated with LPS, respectively. (**C**) Individual values of biological replicates for the −LPS and +LPS conditions. Dashed lines represent the luminescence values for the 0 pg/mL standard and the two lowest IL-1β standard concentrations. N = 3. **** *p* < 0.0001.

We first quantified IL-1β levels under basal and LPS-stimulated conditions (10 ng/mL, 24 h; Figure 2A) using a bioluminescence-based immunoassay (Lumit^®^ IL-1β Mouse Immunoassay, Promega Corporation, Madison, WI, USA). Compared to conventional ELISAs, this assay offers streamlined workflow with reduced time-to-result while maintaining high sensitivity and a broad dynamic range (11 pg/mL–20 ng/mL). A standard curve showed a strong fit to a second-degree polynomial regression (R^2^ = 0.9997; Figure 2B). Analysis of BMM supernatants showed increased IL-1β following LPS stimulation (mean 36.3 pg/mL vs. 3 pg/mL in controls; Figure 2C). Although these values fell near the lower end of the assay’s dynamic range, the corresponding standards also demonstrated a strong fit with the regression model (R^2^ = 0.9868; Figure 2B). However, IL-1β levels in the untreated controls were below the levels of the lowest IL-1β standard, which may complicate interpretation when evaluating inhibitory or protective effects in a scenario of low-grade inflammation (Figure 2C).

#### 2.1.2. Cell-Based Functional Assay

**Figure 3:**
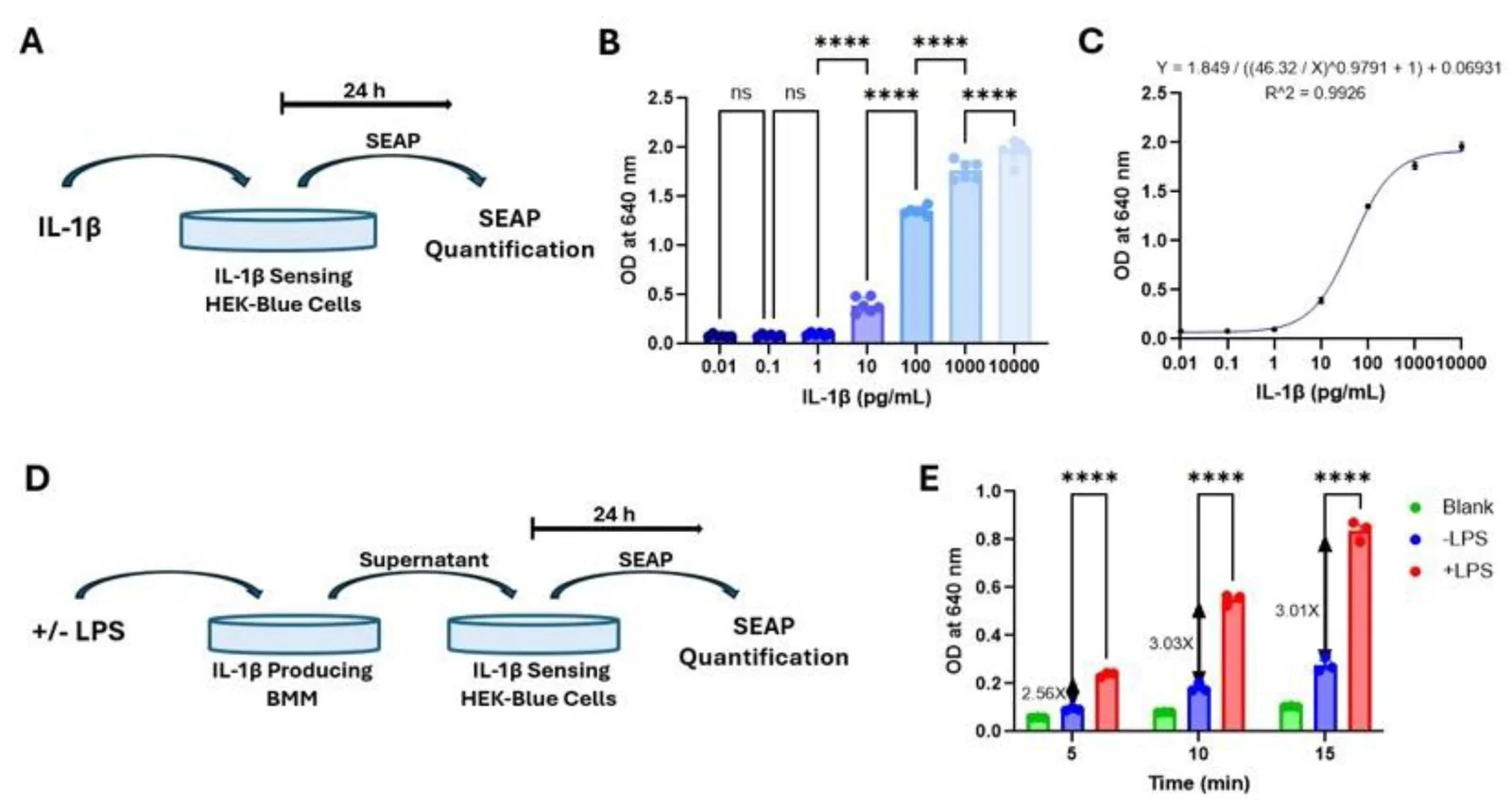
Evaluation of the HEK-Blue IL-1R reporter platform for IL-1β detection. (**A**) Experimental protocol used to assess the dynamic range in response to IL-1β. (**B**) Statistical comparison among IL-1β standards ranging from 0.01 to 10,000 pg/mL as measured by the HEK-Blue IL-1R system. (**C**) Nonlinear four-parameter logistic (4PL) regression model generated from IL-1β standards. (**D**) Experimental workflow to obtain IL-1β-containing supernatants from BMMs in the presence or absence of LPS (10 ng/mL) and SEAP quantification using the HEK-Blue IL-1R assay. (**E**) Detection of SEAP activity over the course of a 5–15 min incubation with QUANTI-Blue reagent. N = 3, **** *p* < 0.0001, ns = not significant.

HEK-Blue™ IL-1 cells are engineered HEK293 cells that stably express the human IL-1R coupled with an NF-κB/AP-1-inducible SEAP reporter that enables the quantification of IL-1R signaling in response to biologically active IL-1α and IL-1β [34,35,36]. To assess the dynamic range of this platform, HEK-Blue cells were incubated with IL-1β standards ranging from 10^−2^ to 10^5^ pg/mL for 24 h and SEAP in supernatants was quantified using QUANTI-Blue^TM^ reagent (Figure 3A). Optical densities (ODs) from the 10^−2^ and 10^−1^ pg/mL standards were below reliable detection. However, SEAP output increased significantly across the 1–10,000 pg/mL range (Figure 3B). The data showed an excellent fit to a four-parameter logistic (4PL) regression (R^2^ = 0.9926), with the 10–100 pg/mL interval corresponding to the steep, near-linear portion of the sigmoid curve (Figure 3C).

Next, we exposed HEK-Blue cells to supernatants from BMM cultured with or without LPS (10 ng/mL, 24 h). Cells were incubated with medium containing 25% BMM supernatant for 24 h, and supernatants were collected for SEAP quantification using QUANTI-Blue reagent (Figure 3D). Optical density (OD) was measured at 5, 10, and 15 min, with media-only wells used as blanks. As expected for an enzymatic readout, the OD increased over time in both experimental groups but remained at low background levels in the blanks. At all timepoints, supernatants from HEK-Blue cells exposed to media from the LPS-treated BMMs exhibited significantly higher OD values than the controls, and the relative difference between groups remained consistent across the 5–15-min measurement interval (Figure 3E). The intermediate 10 min timepoint was selected as the incubation period for subsequent experiments.

### 2.2. Structure-Dependent Inhibition of NLRP3 by Flavonoids

After establishing the validity of the HEK-Blue-IL-1R platform under low-level inflammatory conditions, we used this approach, in combination with Western blot analysis, to compare the effects of three structurally related flavonoids on NLRP3-associated inflammatory signaling: the polymethoxylated aglycone (NOB), the polyhydroxylated aglycone (QUER), and the glycosylated flavonol (RUT) (Figure 1).

#### 2.2.1. Bioactive IL-1β Levels

**Figure 4:**
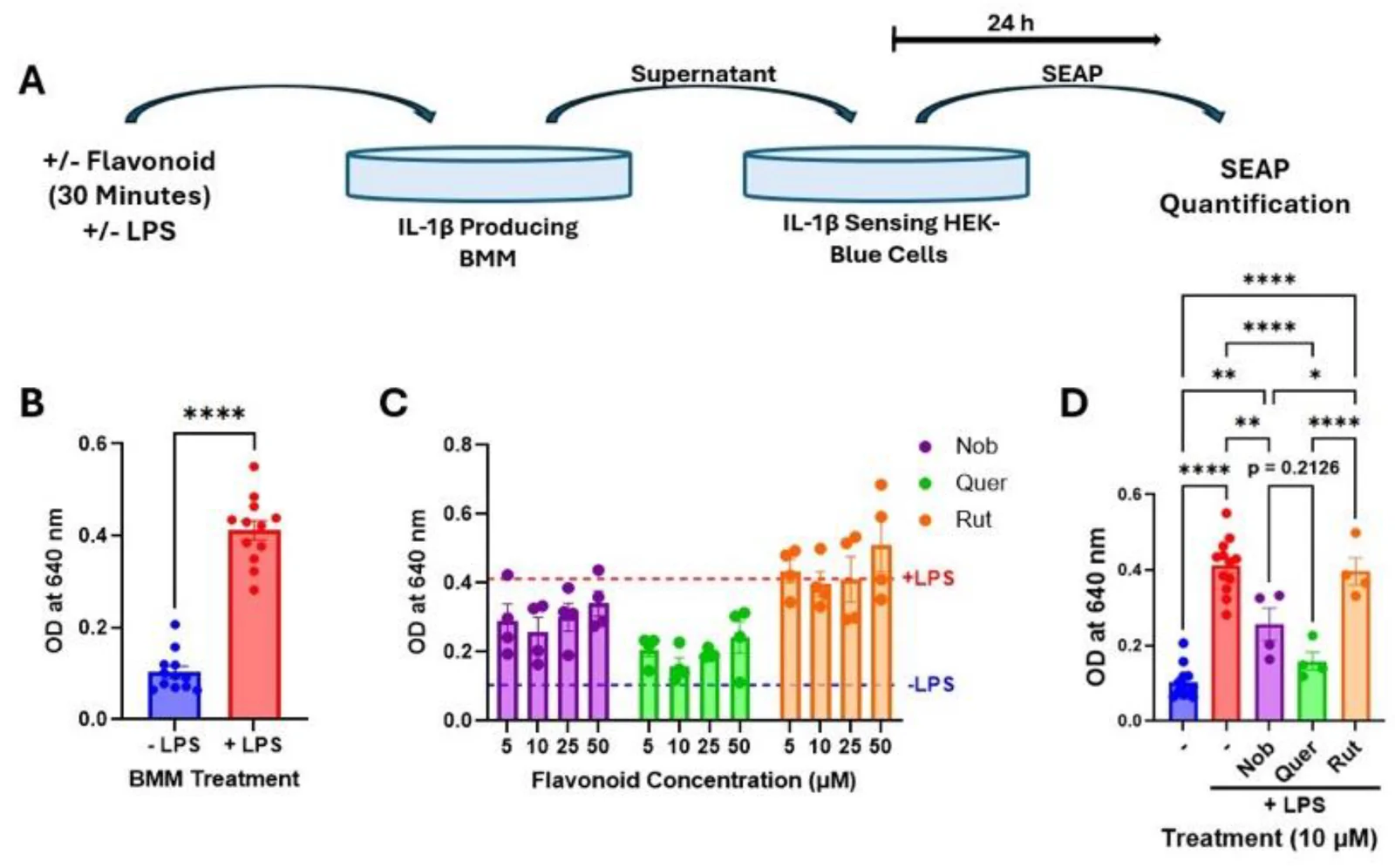
Application of the IL-1R HEK-Blue platform to compare the effects of flavonoids. (**A**) Experimental workflow for the detection of IL-1β in supernatants from BMMs treated with flavonoids and/or LPS using IL-1R HEK-Blue cells. (**B**) Comparison of SEAP activity, measured as OD at 640 nm, in Hek-Blue supernatants following exposure to media from BMMs cultured with or without LPS (10 ng/mL, 24 h). (**C**) Effects of flavonoid pretreatment (5–50 µM; NOB, QUER, or RUT) on SEAP induction by supernatants from LPS-treated BMMs. The −LPS and +LPS dashed lines indicate the mean OD values at 10 min from panel (**B**). (**D**) Comparison of OD values in Hek-Blue cell supernatants following exposure to supernatants from BMMs pretreated with flavonoids (10 µM). N = 12 for the −LPS and + LPS treatment groups and n = 4 for the flavonoid pretreatment groups. * *p* < 0.05, ** *p* < 0.01, **** *p* < 0.0001.

BMMs were either left untreated or pretreated for 30 min with NOB, QUER, or RUT (5, 10, 25, or 50 µM). Cells were subsequently stimulated with LPS (10 ng/mL, 24 h) in the continued presence of the flavonoids. Supernatants were collected and added to HEK-Blue cells as 25% BMM supernatant in culture media. Following a 24 h incubation, culture media were harvested for the quantification of SEAP activity (Figure 4A). As expected, LPS stimulation produced a significant ~4-fold increase in SEAP (Figure 4B). Both aglycone flavonoids significantly reduced SEAP activity, with maximal inhibition observed at 10 µM (Figure 4C). Across all tested concentrations, the polyhydroxylated QUER exhibited greater inhibitory activity than NOB. At 10 µM, QUER reduced LPS-induced SEAP activity by more than 80%, whereas NOB produced approximately 50% inhibition (Figure 4C,D). However, differences between NOB and QUER were not statistically significant based on ANOVA with post hoc analysis (Figure 4D). In contrast to the aglycones, pretreatment with RUT did not affect SEAP activity at any of the concentrations tested (Figure 4C,D).

**Figure 5:**
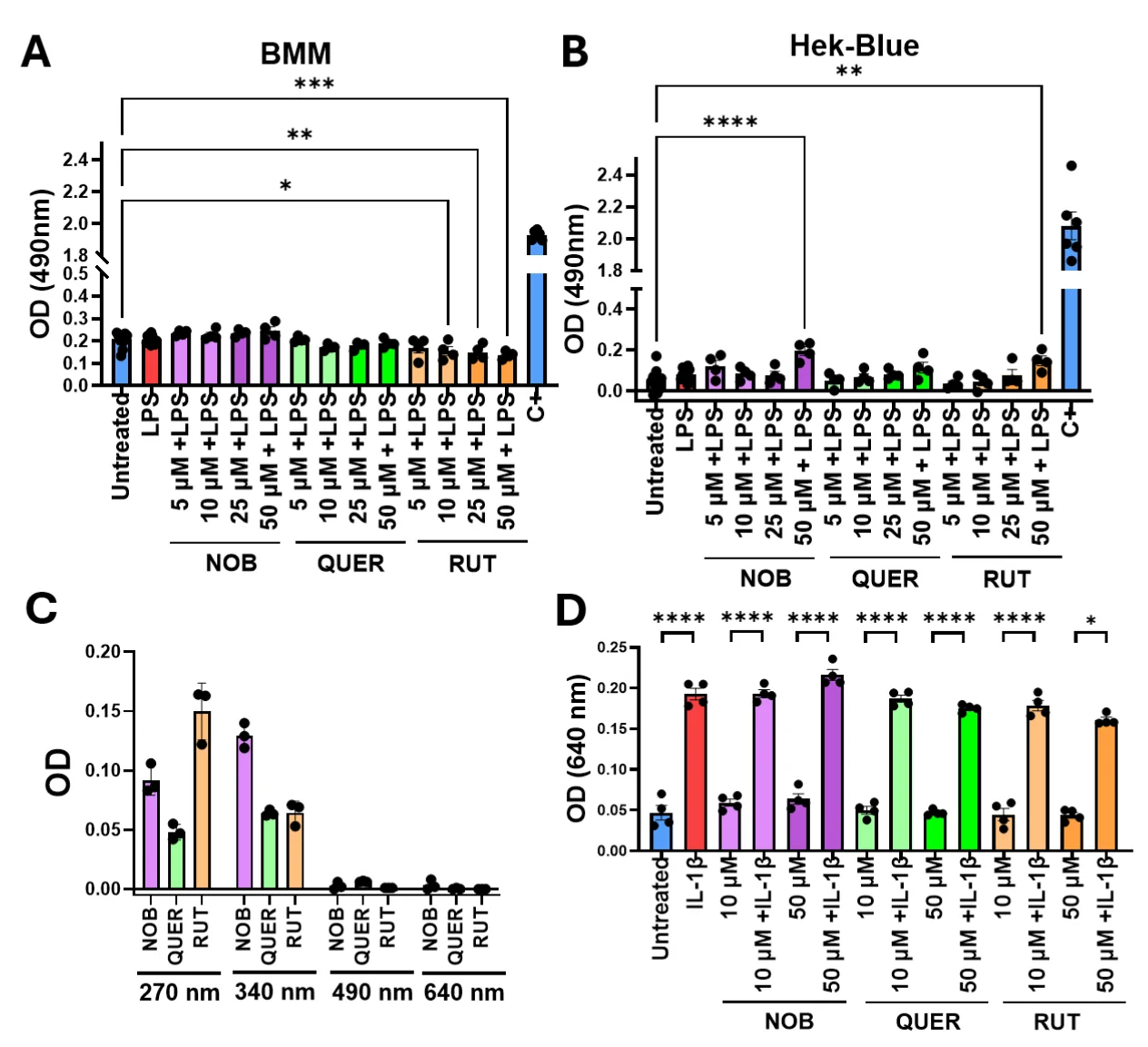
Quality controls for potential effects on cell viability and the Hek-Blue reporter platform. (**A**,**B**) LDH measurements in supernatants of BMM (**A**) and Hek-Blue cells (**B**) used in the experiments shown in Figure 4. (**C**) Absorbance of flavonoid solutions (50 µM in water) measured at the indicated wavelengths (n = 3). (**D**) Hek-Blue cells were incubated with recombinant IL-1β (10 pg/mL, 24 h) in the presence of 25% media containing either no flavonoids or flavonoids at 10 or 50 µM. SEAP production was quantified using QUANTI-Blue (n = 4). * *p* < 0.05, ** *p* < 0.01, *** *p* < 0.001, **** *p* < 0.0001.

The reduced SEAP activity in flavonoid-treated groups could be related to indirect effects on macrophage or HEK-Blue cell viability, the effects of residual flavonoids in BMM-conditioned media on NF-κB/AP-1 signaling or SEAP production by Hek-Blue cells, or interference with QUANTI-Blue colorimetric detection. To assess potential effects on cell viability, we quantified lactate dehydrogenase (LDH) in the supernatants of both BMMs and Hek-Blue cells. None of the experimental conditions increased LDH in the BMM supernatants over the untreated controls, and interestingly, LDH was even slightly lower in the cells treated with RUT at 10, 25, or 50 µM (Figure 5A). Exposure to supernatants from BMMs was also well-tolerated by Hek-Blue cells, with only supernatants from BMMs treated with LPS and the highest concentrations of NOB or RUT (50 µM) inducing a slight increase in LDH compared with the untreated controls (Figure 5B). Flavonoids could also potentially contribute directly to the absorbance measured in the colorimetric assays. However, previous studies have shown that flavonoids absorb primarily in the UV range, with the main absorption bands between 240–280 and 300–380 nm [39]. Consistently, 50 µM solutions of all 3 flavonoids used in this study showed absorbance at 270 and 340 nm but not at 640 nm or at 490 nm, the wavelengths used for the QUANTI-Blue and LDH assays, respectively (Figure 5C). To further determine whether flavonoids interfere with the reporter’s response to IL-1β, Hek-Blue cells were cultured with or without recombinant IL-1β (10 pg/mL) in the presence of 25% media containing 10 or 50 µM NOB, QUER, or RUT. IL-1β-induced SEAP production in the presence of either concentration of each flavonoid was comparable to that observed in the absence of flavonoids (Figure 5D).

#### 2.2.2. NLRP3 Inflammasome Protein Levels

**Figure 6:**
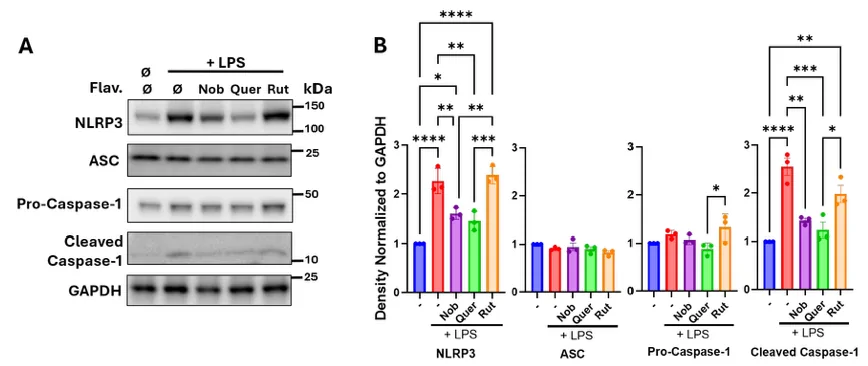
Western blot analysis of inflammasome-related proteins. (**A**) Representative Western blots of NLRP3, ASC, pro-caspase-1, cleaved caspase-1, and GAPDH (loading control) in BMMs treated with ±LPS in the presence or absence of NOB, QUER, or RUT. (**B**) Quantification of integrated band density normalized to the GAPDH loading control and −LPS control. N = 3. * *p* < 0.05, ** *p* < 0.01, *** *p* < 0.001, **** *p* < 0.0001.

Based on the strongest inhibition of IL-1β secretion by flavonoids observed at 10 µM, this concentration and the same culture conditions described above were used to correlate readings from the HEK-Blue reporter system with the immunoblot analysis of NLRP3 inflammasome protein expression and caspase-1 activation. Among the NLRP3 inflammasome core components (NLRP3, ASC, and pro–caspase-1), NLRP3 expression is transcriptionally induced during priming via NF-κB signaling and represents a critical checkpoint for inflammasome activation. In contrast, ASC and pro–caspase-1 are mostly constitutively expressed and are primarily regulated at the level of inflammasome assembly and activation [17,40]. However, modest, context-dependent changes in pro–caspase-1 expression have been reported [40]. BMMs obtained from three individual mice were analyzed independently. Immunoblot analysis with densitometric quantification showed that LPS treatment (10 ng/mL, 24 h) led to a marked induction of NLRP3, while ASC levels remained unchanged, and pro–caspase-1 exhibited a modest tendency toward higher expression (Figure 6A,B and Supplementary Materials). Consistent with the HEK-Blue reporter data, both aglycones significantly inhibited NLRP3 induction and attenuated the modest increase in pro–caspase-1, whereas RUT had no detectable effect on these parameters (Figure 6A,B). Cleaved caspase-1 (p20) was evaluated as a marker of NLRP3 inflammasome activation. Under our experimental conditions, activation levels were low in the LPS-treated cells, consistent with a mild inflammatory response and limited IL-1β secretion. Nevertheless, cleaved caspase-1 levels closely paralleled NLRP3 expression, with significant inhibition by both aglycones (Figure 6A,B).

## 3. Discussion

Low-grade inflammation plays a central role in the development of many chronic diseases, underscoring the need for therapies that are suitable for long-term intervention. Thanks to their established safety profiles and overall biological compatibility, natural compounds are more likely to meet this need than many synthetic xenobiotics. Although natural compounds of the same families share core structural features, there can be significant variability in functional groups that can substantially influence their biological activities. Flavonoids are abundant in natural food sources, and many have demonstrated benefits against chronic diseases through multiple mechanisms, including anti-inflammatory effects [22,29,41,42,43,44]. However, studies investigating structure–effect relationships among flavonoids remain limited. Here, we compared three naturally abundant flavonoids with related flavone/flavonol scaffolds but distinct functional groups: the polymethoxylated aglycone NOB, the polyhydroxylated aglycone QUER, and the glycosylated flavonol RUT [23,24,25]. Using a low-grade inflammation model and complementary reporter and protein expression analyses, we found that NOB and QUER attenuated NLRP3-related responses, whereas RUT had no detectable effect under the conditions tested.

A key challenge studying low-grade inflammation is the accurate measurement of differences in relatively low cytokine levels, particularly when evaluating protective interventions that further reduce cytokine production. We have previously found that prolonged exposure of BMMs to a low concentration of LPS is sufficient to induce NLRP3-related IL-1β secretion within the low-pg/mL range [30]. Although this relatively low IL-1β level may better reflect the modest inflammatory signaling associated with chronic disease than the robust responses typically observed in acute inflammasome models, it presents challenges for conventional quantitative assays. We compared the performance of a high-sensitivity bioluminescence immunoassay with that of a HEK-Blue reporter system that produces SEAP in response to IL-1β signaling. Both approaches detected significant differences in IL-1β production between the untreated and LPS-stimulated macrophages. Although the immunoassay provided a broader quantitative range, its sensitivity was limited near the lower end of detection. In contrast, the HEK-Blue reporter exhibited a narrower linear dynamic range but was well-suited for detecting low-picomolar concentrations of IL-1β and enabled clear discrimination between basal IL-1β secretion and background signal. Importantly, the accompanying viability and interference controls indicated that the experimental conditions were generally well-tolerated by both BMMs and HEK-Blue cells and that the observed changes in SEAP production were unlikely to result from cytotoxicity, direct optical interference, or nonspecific suppression of IL-1R signaling.

The LPS exposure paradigm used in this study differs from the classical two-signal model of acute NLRP3 inflammasome activation [18,45]. Furthermore, an important caveat is that the Hek-Blue reporter responds to both bioactive IL-1β and IL-1α and therefore cannot by itself establish specific NLRP3-dependent IL-1β production. To further relate the findings from the reporter assay to NLRP3 inflammasome activation, we performed Western blot analysis of NLRP3 inflammasome components. LPS-induced NLRP3 expression, as well as its attenuation by NOB and QUER but not RUT, was consistent with the findings from the reporter cell line. The relatively low levels of cleaved caspase-1 observed in the LPS-treated macrophages further indicate that inflammasome activation remained modest under these experimental conditions, consistent with the low-level IL-1 signaling detected with the reporter. Although additional analyses, such as the use of broad or specific inhibitors, assessment of pro-IL-1β and mature IL-1β, secreted cleaved caspase-1, ASC speck formation, or caspase-1 activity assays, could provide greater insight into the specific steps of the inflammasome pathway affected by the flavonoids, the Western blot analysis is consistent with the Hek-Blue reporter results and supports that NOB and QUER, but not RUT, attenuate NLRP3-related inflammatory signaling. Alternative approaches to link Hek-Blue reporter responses to anti-inflammasome activity could include the neutralization of IL-1β or IL-1α using specific antibodies or validation using immunoassays that selectively quantify mature IL-1β. Although these approaches do not provide direct assessments of inflammasome activation, they may be more amenable to large-scale screening studies than immunoblot-based analysis.

Although the difference did not reach statistical significance, QUER appeared to exert a more robust inhibitory effect than NOB, both in the reporter and Western blot analyses. Consistent with these findings, previous studies comparing methoxylated and hydroxylated flavonoids have associated hydroxyl groups with more robust inhibition of inflammatory targets, whereas methoxy groups are generally associated with increased metabolic stability and lipophilicity [46,47,48]. An important consideration is that flavonoids in natural sources are often found as glycosides. Our findings that RUT did not significantly induce inflammasome proteins or IL-1β secretion is consistent with previous reports suggesting that glycosylation reduces intrinsic activity in cell-based systems [49,50]. However, cell culture models do not fully capture the metabolic and physiological complexity of the in vivo environment. Flavonoid glycosides can be hydrolyzed by intestinal enzymes and the gut microbiota, generating the more active aglycone forms prior to or during absorption. Moreover, glycosylation may enhance solubility and stability in the digestive tract, and glycosylated forms of QUER have been associated with higher net absorption than the aglycone [51,52,53,54]. Direct in vivo comparisons between these compounds will therefore be important to better define how flavonoid structure, metabolism, and bioavailability collectively influence their physiological effects. A better understanding of how specific functional groups influence the balance between intrinsic biological activity and pharmacokinetic behavior will be essential for optimizing the therapeutic use of flavonoids and other anti-inflammatory natural compounds. Such considerations are particularly important for chronic inflammatory diseases, where long-term efficacy and tolerability are critical.

## 4. Materials and Methods

### 4.1. Bone Marrow Macrophages

All animal protocols were approved by the Albany Medical College Institutional Animal Care and Use Committee (protocol number 23-11002). C57BL/6J mice originating from The Jackson Laboratory, Bar Barbor, ME, USA were housed under regular 12/12 light–dark cycles, under a regulated temperature of 20–26 °C and humidity level of 30–70%, and fed a standard chow diet Prolab^®^ Isopro^®^ RMH 300-5P76, LabDiet, St. Louis, MO, USA). Bone marrow cells were isolated from three ~8–12-week-old female mice euthanized under isoflurane anesthesia as previously described [30,55,56]. Cells were cultured in 10 cm Petri dishes containing RPMI-1640 media supplemented with 10% heat-inactivated fetal bovine serum (FBS), antibiotic/antimycotic (penicillin 10 μg/mL, streptomycin 10 μg/mL, amphotericin B 0.025 μg/mL), and 15% (*v*/*v*) L929 cell-conditioned medium (LCM). Media were replaced every 48 h, and cells were passaged upon reaching approximately 70% confluence. For experiments, cells were plated at a density of 1 × 10^6^ cells/mL on non-tissue-treated Petri dishes and incubated overnight before treatments. For Western blot studies, the three independent sets of BMMs were analyzed separately. To evaluate the protective effects of flavonoids using the Hek-Blue platform, BMMs were pooled to obtain a homogeneous cell suspension and plated in 96-well Petri dishes. In both experiments, cells were pre-treated with NOB, QUER, or RUT (5, 10, 25, or 50 µM) for 30 min before stimulation with LPS (10 ng/mL) to model low-grade inflammatory conditions. Flavonoids were prepared from 10 mM stock solutions in DMSO and diluted in culture medium immediately before use. Flavonoids remained in the medium throughout the LPS exposure period. After 24 h of LPS treatment, supernatants and cells were collected for immediate analysis or stored at −80 °C.

### 4.2. Bioluminescense-Based Immunoassay

Basal and LPS-induced IL-1β levels were quantified using the Lumit^®^ Mouse IL-1β Immunoassay Kit (Promega Corporation, Madison, WI, USA). A 10 µg/mL IL-1β stock standard was diluted 1:500 in LCM-supplemented RPMI medium to generate a 20 ng/mL working standard. Serial dilutions (2:7) were subsequently prepared to obtain standards at concentrations of 1.63, 0.47, 0.13, 0.038, and 0.011 ng/mL. BMMs were treated with 10 ng/mL LPS or left untreated, and culture supernatants collected after overnight incubation were used as samples. The antibody working solution was prepared by mixing 8 µL anti-mIL-1β mAb-SmBiT and 8 µL anti-mIL-1β mAb-LgBiT with 4 mL LCM-supplemented RPMI medium. In the presence of IL-1β, the SmBiT and LgBiT luciferase subunits reconstitute an active luciferase enzyme that reacts with the substrate to generate a luminescent signal proportional to IL-1β concentration. Standards and samples were added to a 96-well protein-binding plate, followed by the addition of antibody solution and substrate according to the manufacturer’s instructions. Luminescence was measured after a 5 min incubation period using a BioTek Cytation5 imaging reader (Agilent Technologies, Santa Clara, CA, USA).

### 4.3. Human Embryonic Kidney IL-1β Reporter Cells

HEK-Blue IL-1R reporter cells were obtained from InvivoGen (San Diego, CA, USA). These cells contain a modified IL-1β signaling pathway in which binding of IL-1α or IL-1β to IL-1R induces the transcription and translation of a secreted embryonic alkaline phosphatase (SEAP) reporter gene. Cells were thawed and cultured in 10 cm tissue-culture-treated dishes containing high-glucose DMEM supplemented with 10% FBS and antibiotic/antimycotic (penicillin 10 μg/mL, streptomycin 10 μg/mL, amphotericin B 0.025 μg/mL). Media were replaced every 48 h, and cells were passaged upon reaching approximately 70% confluence. For experiments, HEK-Blue IL-1R cells were seeded at a density of 3 × 10^5^ cells/mL on tissue-culture-treated plates and treated either with recombinant IL-1β or BMM-conditioned media diluted to 25% in culture media. Following 24 h incubation at 37 °C, SEAP-containing supernatants were collected for analysis.

### 4.4. QUANTI-Blue SEAP Detection

SEAP levels, used as an indirect measure of IL-1β activity in the supernatant, were quantified using a colorimetric enzyme assay with QUANTI-Blue solution (InvivoGen, San Diego, CA, USA). QUANTI-Blue working solution was prepared by mixing QB reagent, QB buffer, and Milli-Q water at a 1:1:98 ratio, followed by vortexing and incubation at room temperature for 10 min. For the assay, 25 µL of HEK-Blue cell supernatant was added to each well of a 96-well plate, followed by 225 µL of QUANTI-Blue solution. Samples were mixed by pipetting and incubated at 37 °C. OD was measured spectrophotometrically at 640 nm every 5 min until signal saturation was reached.

### 4.5. Cell Viability Assays

Supernatants from BMM and Hek-Blue cells stored at −80 °C were assessed for cytotoxicity using the CyQUANT™ LDH Cytotoxicity Assay (Thermo Fisher Scientific, Waltham, MA, USA). Samples were assayed using freshly prepared reagents according to the manufacturer’s instructions, in parallel with an LDH positive control. Following a 30-min incubation in the dark at room temperature, net 490 nm absorbance was used to compare LDH release among experimental groups.

### 4.6. SDS-PAGE and Western Blotting

BMMs obtained from three individual mice were analyzed independently. Protein samples were isolated using radioimmunoprecipitation assay (RIPA) buffer supplemented with a protease inhibitor. Total protein was resolved by SDS-PAGE and transferred to a nitrocellulose membrane. Pre-cast 4–20% polyacrylamide gels were used for NLRP3, ASC, pro–caspase-1, and GAPDH, and 10–20% pre-cast polyacrylamide gels were used for cleaved caspase-1. Following the transfer, membranes were incubated with primary antibodies followed by HRP-conjugated secondary antibodies. The primary antibodies and dilutions used were: M-α-NLRP3 1:1000 (Adipogen Life Sciences, San Diego, CA, USA), G-α-ASC 1:1000 (Cell Signaling Technology, Danvers, MA, USA), M-α-Caspase 1 1:1000 (Adipogen Life Sciences, San Diego, CA, USA), R-α-Cleaved Caspase-1 1:1000 (Cell Signaling Technology, Danvers, MA, USA), and R-α-GAPDH 1:10,000 (Sigma-Aldrich, St. Louis, MO, USA). Protein bands were visualized using a chemiluminescent substrate followed by imaging. Images were analyzed using ImageJ software (v1.5gJ), and signal intensity was quantified by measuring the integrated density of treatment bands relative to the −LPS control. Background intensity was measured directly beneath each protein band and subtracted from the corresponding band intensity to calculate the net signal intensity. GAPDH was used as a housekeeping gene to normalize protein concentration.

### 4.7. Statistical Analyses

Data are presented as mean ± SEM from a minimum of three biological replicates. Statistical analyses and graph generation were performed using Prism software version 10 (GraphPad), with statistical significance defined as *p* < 0.05. Standard curves for the immunoassay and HEK-Blue assays were generated using second-degree polynomial regression (Y = 8.622X − 0.0004892X^2^ + 6.798) and four-parameter logistic (4PL) regression (Y = 1.849/((0.04632/X)^0.9791^ + 1) + 0.06931), respectively. Normality was assessed using the Shapiro–Wilk test. For pairwise comparisons, data that followed a normal distribution using two-tailed *t*-test and data that did not follow a normal distribution were analyzed using the two-tailed Mann–Whitney U. Statistical significance for HEK-Blue IL-1β dynamic range, cytotoxicity assays, and Western blot band intensity analyses was assessed using one-way ANOVA followed by Tukey’s or Dunnett post hoc tests. Flavonoid dose–response analyses using the HEK-Blue reporter assay were evaluated by two-way ANOVA with Tukey’s multiple comparisons test, comparing treatments within each dosage group.

## 5. Conclusions

Chronic inflammation remains a major clinical challenge that requires therapies suitable for long-term use. This study evaluated a reporter cell line as an alternative to immunoassays for comparing the effects of structurally distinct flavonoids on IL-1β secretion by macrophages. The HEK-Blue-IL-1R reporter system demonstrated good sensitivity and linearity at low cytokine concentrations and effectively discriminated weak inflammatory signals from background activity. The reporter responses were consistent with the Western blot analysis of inflammasome-associated proteins, supporting the utility of this platform for detecting changes in bioactive IL-1β in the context of low-grade inflammation. Both experimental approaches supported anti-inflammasome activity of the flavonoid aglycones NOB and QUER, whereas the glycosylated flavonol RUT had no detectable effect under the conditions tested. Although the difference between NOB and QUER did not reach statistical significance, QUER showed a modestly greater inhibitory effect, consistent with the potential contribution of hydroxylation to flavonoid anti-inflammatory activity. Given that chronic inflammatory conditions such as metabolic and cardiovascular diseases are associated with relatively low circulating concentrations of IL-1β, which may approach the lower detection limits of conventional immunoassays [57,58,59], the HEK-Blue-IL-1R reporter platform may provide a sensitive and efficient approach for screening compounds and therapeutic strategies targeting inflammasome-associated inflammatory signaling.

## Data Availability

The datasets generated during and/or analyzed during the current study are available from the corresponding author on reasonable request.

## Supplementary Materials

The following supporting information can be downloaded at: https://www.mdpi.com/article/10.3390/molecules31183176/s1, Original Western blot images of Figure 6A and replicate blots.

## Abbreviations

The following abbreviations are used in this manuscript:

AP-1	Activator Protein 1
ASC	Apoptosis-Associated Speck-Like Protein Containing a CARD
CARD	Caspase Activation and Recruitment Domain
DAMP	Damage-Associated Molecular Pattern
DMEM	Dulbecco’s Modified Eagle Medium
DMSO	Dimethyl Sulfoxide
FBS	Fetal Bovine Serum
HEK	Human Embryonic Kidney
LCM	L929 Conditioned Medium
NF-κB	Nuclear Factor Kappa B
NLRP3	NLR Family Pyrin Domain Containing 3
NSAID	Nonsteroidal Anti-Inflammatory Drug
PAMP	Pathogen-Associated Molecular Pattern
QB	QUANTI-Blue
SEAP	Secreted Embryonic Alkaline Phosphatase
